# Vitamin C uncouples the Warburg metabolic switch in KRAS mutant colon cancer

**DOI:** 10.18632/oncotarget.10087

**Published:** 2016-06-15

**Authors:** Oscar Aguilera, María Muñoz-Sagastibelza, Blanca Torrejón, Aurea Borrero-Palacios, Laura del Puerto-Nevado, Javier Martínez-Useros, María Rodriguez-Remirez, Sandra Zazo, Estela García, Mario Fraga, Federico Rojo, Jesús García-Foncillas

**Affiliations:** ^1^ Cancer Biomarkers Research Group, Fundacion Jimenez Diaz University Hospital Health Research Institute, UAM, 28040 Madrid, Spain; ^2^ Translational Oncology Division, Oncohealth Institute, Fundacion Jimenez Diaz University Hospital, 28040 Madrid, Spain; ^3^ Cancer Epigenetics Laboratory, Instituto Universitario de Oncología del Principado de Asturias (IUOPA-HUCA), Universidad de Oviedo, 33011 Oviedo, Spain

**Keywords:** colon cancer, Warburg, vitamin C, GLUT-1

## Abstract

*KRAS* mutation is often present in many hard-to-treat tumors such as colon and pancreatic cancer and it is tightly linked to serious alterations in the normal cell metabolism and clinical resistance to chemotherapy.

In 1931, the winner of the Nobel Prize in Medicine, Otto Warburg, stated that cancer was primarily caused by altered metabolism interfering with energy processing in the normal cell. Increased cell glycolytic rates even in the presence of oxygen is fully recognized as a hallmark in cancer and known as the Warburg effect.

In the late 1970′s, Linus Pauling and Ewan Cameron reported that vitamin C may have positive effects in cancer treatment, although deep mechanistic knowledge about this activity is still scarce.

We describe a novel antitumoral mechanism of vitamin C in *KRAS* mutant colorectal cancer that involves the Warburg metabolic disruption through downregulation of key metabolic checkpoints in *KRAS* mutant cancer cells and tumors without killing human immortalized colonocytes.

Vitamin C induces RAS detachment from the cell membrane inhibiting ERK 1/2 and PKM2 phosphorylation. As a consequence of this activity, strong downregulation of the glucose transporter (GLUT-1) and pyruvate kinase M2 (PKM2)-PTB dependent protein expression are observed causing a major blockage of the Warburg effect and therefore energetic stress.

We propose a combination of conventional chemotherapy with metabolic strategies, including vitamin C and/or other molecules targeting pivotal key players involved in the Warburg effect which may constitute a new horizon in anti-cancer therapies.

## INTRODUCTION

The KRAS proto-oncogene encodes a ~21 kDa small GTPase, which cycles between GTP-bound active and GDP-bound inactive states. Mutated KRAS is reported in approximately 35%-45% of colorectal cancers and >90% of pancreatic ductal adenocarcinoma (PDAC) [1, 2]. Molecular evidences support the master role of oncogenic KRAS disrupting the metabolic homeostasis via alteration of glucose uptake, glycolytic flux, and glutamine usage in colon and pancreatic tumors that often display very high resilience to chemotherapy [3].

Quest for new scopes and molecules capable to overcome chemotherapy resistance in tumors displaying gene mutations downstream EGFR is a top priority in oncological research worldwide.

In 1976, Linus Pauling and Ewan Cameron performed a clinical study of the survival times of 100 terminal cancer patients who were given supplemental ascorbate, usually 10 g/day, and 1000 matched controls, similar patients who had received the same treatment except for the ascorbate. Survival times greater than 1 yr after the date of untreatability were observed for 22% of the ascorbate-treated patients and for 0.4% of the controls [4].

Many authors have reported that vitamin C shows certain antitumoral activity, but the molecular mechanism underlying this killing effect and the intriguing selective activity displayed is far from clear.

Recently, Yun J *et al*., have recently presented noteworthy data stating that oxidized vitamin C is able to kill CRC cells depending on the *KRAS* mutational status [5]. Interestingly, previous works carried out by Chen Q *et al*., supporting the previous clinical study carried out by Pauling and Cameron, have shown that vitamin C exerts killing effects on cancer cells from very different origin, displaying a wide variety of gene mutations (many of them do not display KRAS mutation) and alterations in different signaling pathways [1, 6]. It strongly suggests that although *KRAS* status is important to explain the killing effect of vitamin C, there must be other mechanisms underlying its role in cancer.

Hanahan and Weinberg [7], stated that six essential hallmarks in cell physiology could enhance malignant cell growth 1) self-sufficiency in growth signals, 2) insensitivity to growth inhibitory signals, 3) evasion of programmed cell death (apoptosis), 4) limitless replicative potential, 5) increased vascularity (angiogenesis), and 6) tissue invasion and metastasis.

However, another abnormality in cell homeostasis is currently considered as the seventh hallmark in cancer.

Biochemist Otto Warburg received the Nobel Prize in 1931 showing that, contrary to normal cell metabolism, which primarily rely on mitochondrial oxidative phosphorylation to generate ATP, most cancer cells show increased glycolysis rate. This phenomenon is termed the Warburg effect and it is a hallmark in cancer [8].

Nevertheless, the hypothesis that rocked the scientific community was his assertion that the prime cause of cancer is the replacement of the oxygen respiration in normal body cells by a fermentation of sugar [9].

Warburg hypothesized that cancer was caused by defects in mitochondrial oxidative phosphorylation and then forcing the cell to switch into glycolysis, thus cells would become undifferentiated and cancerous.

However, studies carried out by prof. Craig Thompson laboratory at the Memorial Sloan-Kettering Cancer Center, preferentially indicates that the Warburg effect is not just a passive response to damaged mitochondria but results from oncogene-directed metabolic reprogramming required to support glycolytic metabolism and anabolic growth [10].

The question about altered metabolism as primary cause or consequence in cancer still remains open.

Alterations of EGFR/MAPK signaling are frequently observed in *KRAS* and *BRAF* colon cancer correlating with chemoresistance and poor clinical outcome. Furthermore, mutations in EGFR/MAPK pathway and associated resistance to anti-EGFR therapies are, in fact, linked to the metabolic alterations described by Otto Warburg in cancer cells.

In this regard, Makinoshima H *et al.,* published data showing that epidermal growth factor receptor (EGFR) signaling actually regulates global metabolic pathways in EGFR-mutated lung adenocarcinoma [11]. They demonstrated that EGFR-tyrosine kinase inhibitors (TKIs) were able to decrease lactate production, glucose consumption, and the glucose-induced extracellular acidification rate (ECAR), indicating that EGFR signaling was responsible for maintaining cell aerobic glycolysis observed in the Warburg effect. As it has been stated before, molecular evidences strongly support the role of oncogenic KRAS disrupting the normal cell metabolism in tight correlation to tumor resilience to anti-EGFR chemotherapy [1].

Anti-cancer strategies based on molecules targeting crucial enzymes involved in tumoral aerobic glycolysis, may help to overcome anti-EGFR resistance in cancer improving the response of those patients to conventional chemotherapy.

Here we present data describing a novel antitumoral mechanism of vitamin C that involves straight inhibition of constitutively activated EGFR/MAPK pathway in *KRAS* mutant CRC, which in turn provokes the stalling of the Warburg metabolism.

## RESULTS AND DISCUSSION

### Vitamin C selectively kills KRAS mutant colon cancer cells alone or in combination with cetuximab

We aimed to check out whether vitamin C could have some antitumoral activity in chemoresistant CRC in assays carried out using SW480 and LoVo cell lines, displaying *KRAS* mutations and no sensitivity to cetuximab.

**Figure 1:**
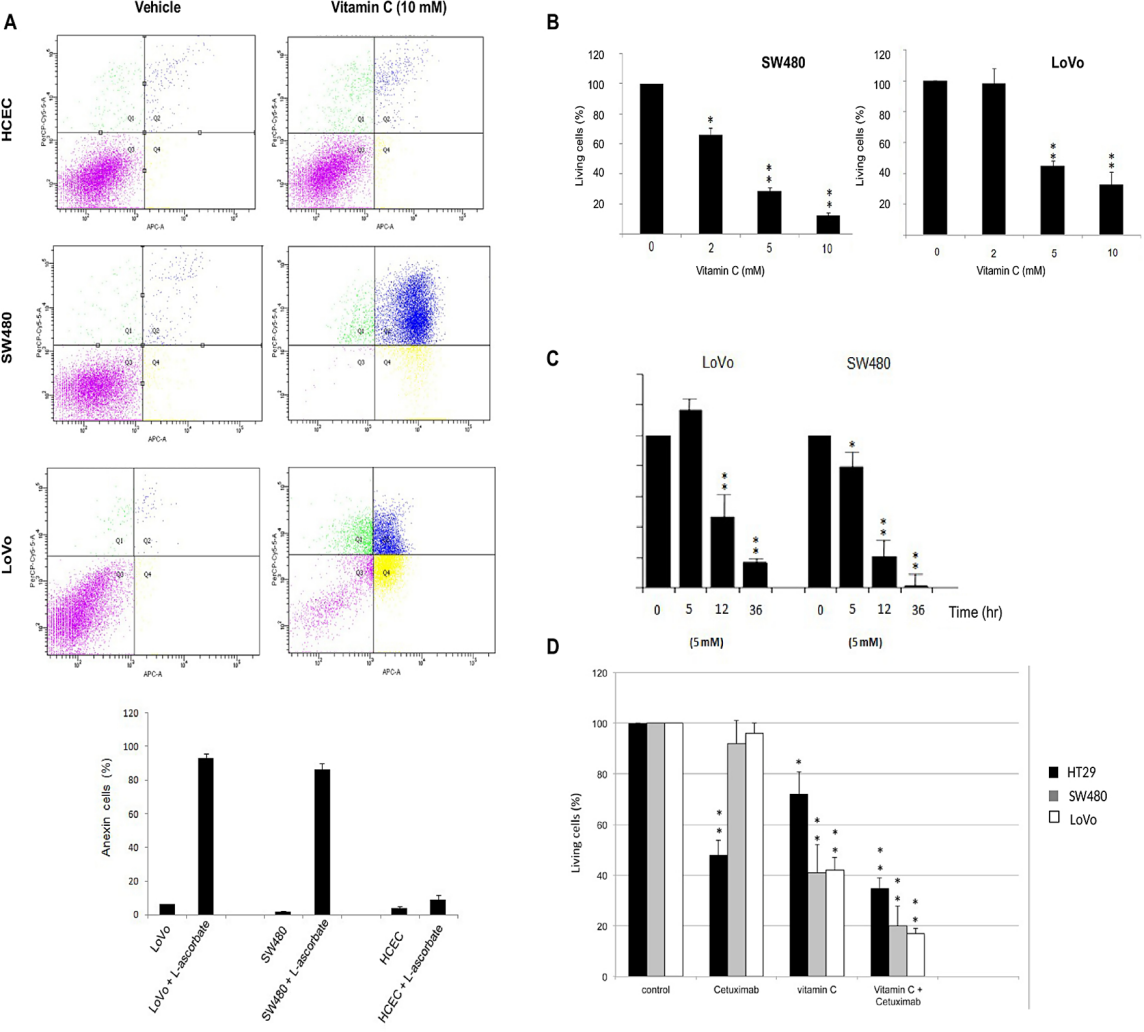
Vitamin C selectively kills wild type and mutant *KRAS* colon cancer cells alone or in combination with cetuximab **A.** apoptosis-inducing activities of vitamin C for Normal human immortalized Colonocytes (HCEC) and SW480 and LoVo cancer cell lines were annalyzed by Annexin-PI assay. Each cell line (2,3 10^5^ cells) was incubated with vitamin C (10 mM) and PBS for controls. for 20 hr, apoptosis in each cell line was measured by staining with FITC-conjugated Annexin-V and Propidium Iodide (PI) using a Sigma-Aldrich Apoptosis kit. The populations of cells (annexin-V positive/PI negative) and late apoptotic cells (PI positive) as a percent of total cells were evaluated. Vitamin C displayed a selective killing effect on SW480 and LoVo. One-way ANOVA followed by Dunnett's post-test for multiple comparisons. *p< 0.05, **p < 0.001, n = 3. **B.** vitamin C treatment at different concentrations were carried out with LoVo and SW480 cancer lines. SW480 and LoVo CRC lines were exposed to ascorbate at 2, 5 and 10mM, for 20 hr. Then, cells were tripsinized and fixed with trypan blue solution (Sigma-Aldrich). The effective concentration that decreased survival 50% (EC50) was determined. EC50 was <10 mM for both tumor cells tested. Cell counting was carried out using a TC20™ Automated Cell Counter (Biorad). One-way ANOVA followed by Dunnett's post-test for multiple comparisons. *p < 0.05, **p < 0.001, n = 3. **C.** SW480 and LoVo cells were treated with vitamin C (7mM) for 5, 12 and 36 hr. Then, cells were tripsinized and fixed with trypan blue solution (Sigma-Aldrich). Cell counting was carried out using a TC20™ Automated Cell Counter (Biorad). One-way ANOVA followed by Dunnett's post-test for multiple comparisons. *p < 0.05, **p < 0.001, n = 3. **D.** HT29 harboring wild type *KRAS*, and the *KRAS* mutants LoVo and SW480 were treated with cetuximab (calculated IC50=0,4 μM), vitamin C (5mM) alone and combination for 12 hr. Then, cells were tripsinized and fixed with trypan blue solution (Sigma-Aldrich). Cell counting was carried out using a TC20™ Automated Cell Counter (Biorad). One-way ANOVA followed by Dunnett's post-test for multiple comparisons. *p < 0.05, **p < 0.001, n = 3. Combination of both vitamin C and the anti-EGFR antibody cetuximab displayed a higher killing effect in the three lines tested.

SW480, LoVo cancer cells both harboring *KRAS* mutation (G12V and G13D respectively) and immortalized human colonocytes (HCEC) (*KRAS* wild type) were exposed to ascorbate for 2 h to mimic clinical pharmacokinetics, and the effective concentration that decreased survival to 50% (EC50) was determined. Observed EC50 was ≤ 10mM for both tumor cells lines tested. Remarkably, significative cytotoxicity in HCEC cells treated with 10 mM ascorbate was not detected (Figure 1A–1B). Mortality observed for both cancer cell lines was ≥ 60% just 36 h. after treatment (Figure 1C), although HT29 displayed more resistance to ascorbate.

We conclude that the effect of vitamin C seems to be, at least in part, dependent on the mutational status of *KRAS* as previously stated by Yun J *et al*. [5]. Interestingly, KRAS mutation has been demonstrated to drive aberrant changes in the cell metabolic homeostasis in colon and pancreatic tumors [12, 13].

Therefore, assuming that vitamin C is targeting cells displaying abnormal metabolism, it might help to overcome anti-EGFR monoclonal antibody cetuximab (Erbitux) resistance in tumors harbouring mutated KRAS.

In order to demonstrate this hypothesis we used colon cancer cell lines HT29 (wild type KRAS), LoVo and SW480 (both displaying different *KRAS* mutations) were treated with cetuximab (HT29 calculated IC_50_=0,4 μM). As described, mutations in codon 12 or 13 in the KRAS gene downstream of the EGFR induce constitutive activation of the RAS/RAF/MAPK pathway linked to resistance to anti EGFR therapies. Nevertheless, a retrospective study reported partial response to cetuximab in patients with KRAS G13D mutation [14]. As expected, neither LoVo nor SW480 cells did exhibit any sensitivity to cetuximab (Figure 1D). However, wild type KRAS HT29 cell growth was clearly inhibited by cetuximab to 52%.

The three CRC cell lines tested displayed differential sensitivity to pharmacological concentration of vitamin C (5mM). Again, HT29 colon cancer cells appeared to be more resistant to the cytotoxic activity of vitamin C than SW480 and LoVo.

Remarkably, treatment of HT29, SW480 and LoVo cells with cetuximab (0,4 μM) and vitamin C (5mM) abolished cell growth in the three lines tested. Combination of both, cetuximab and vitamin C was more effective than vitamin C alone, strongly suggesting that vitamin C may synergize with cetuximab via some molecular mechanism not yet described but likely involving metabolic targeting.

In order to support the *in vitro* data, we generated SW480 xenografts harboring KRAS mutation (G12V) in athymic nude mice Foxn1nu. Mice were treated with intraperitoneal (i.p) vitamin C at pharmacological concentration (4gr/kg body weight) once daily. Remarkably, *in vivo* experiments showed that using parenteral ascorbate as unique treatment, tumor growth was strongly reduced by 49 % (P = 0.007) (Figure 4B) thus supporting data obtained in our *in vitro* assays.

Some CRC patients displaying the KRAS G13D mutation may respond to cetuximab, so the question whether combinative dosage of vitamin C plus cetuximab could be even more effective in this type of tumors would need further research.

### MAPK/EGFR pathway is selectively inhibited by vitamin C in KRAS mutant colon cancer

The observation that HT29 with wild type *KRAS* was less sensitive to vitamin C activity than the *KRAS* mutant cell lines SW480 and LoVo encouraged us to further examine the mechanism underlying putative interferences of vitamin C on MAPK/EGFR signaling pathway in both cetuximab-resistant cell lines.

Activated Extracellular-signal-Regulated Kinase 1/2 (ERK1/2) is the key effector of the pathway. In fact, evidences that MAPK/ERK signaling induces cell proliferation, survival and cancer spreading, along with extreme frequency in which this pathway is aberrantly activated in cancer, support current investigations to identify new targets to inhibit ERK activation and other pathway intermediates [15].

*KRAS*/*BRAF* mutation status and also ERK1/2 activation have been recently reported as biomarkers for chemoresistance in gastrointestinal cancer to agents such as 5-Fu, SN38 and Oxaliplatin [16]. Furthermore, activation of ERK1/2 is also responsible of induced chemoresistance of glioma cells to Temozolomide [17].

**Figure 2:**
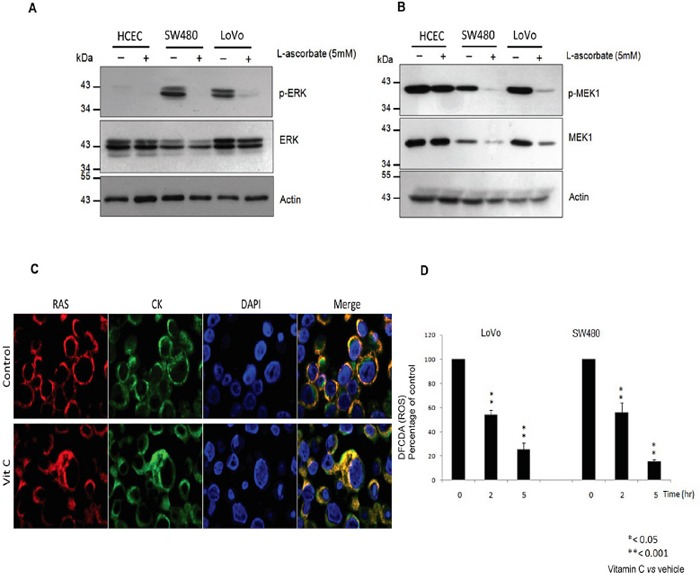
Vitamin C hampers the EGFR/MAPK signaling pathway through *KRAS* detachment from the cell plasma membrane **A.** western blot analysis of ERK1/2 and p-ERK1/2 expression in Normal Human immortalized Colonocytes (HCEC) and the *KRAS* mutant cell lines SW480 and LoVo treated with vitamin C (8 mM) for 20 hr. Beta-actin was employed as protein loading control. **B.** Western blot analysis of MEK1 and p-MEK-1 expression in Normal Human immortalized Colonocytes (HCEC) and the *KRAS* mutant cell lines SW480 and LoVo treated with vitamin C for 20 hr. **C.** LoVo cells were treated with treated with vitamin C for 20 hr. Double immunofluorescence with anti RAS (red) and cytokeratin 20 (green) raised antibodies shows cytoplasmic colocalization of both proteins after vitamin C treatment (8 mM). **D.** activity of vitamin C in quenching intracellular reactive oxygen species (ROS) was measured using a Cellular ROS/Superoxide Detection Assay Kit (Abcam). SW480 and LoVo CRC lines were exposed to ascorbate (7 mM) and PBS for controls for 20 hr. After treatment, cells were washed twice with PBS solution and then incubated in medium containing 10 μmol/l H2DCF-DA at 37°C for 30 minutes. After incubation, cells were harvested by trypsinization, and intracellular levels of ROS were measured by fluorescence emission. Relative intracellular ROS levels after treatment with vitamin C were calculated in relation to control, which was given a value of 100. One-way ANOVA followed by Dunnett's post-test for multiple comparisons. *p < 0.05, **p < 0.001, n = 3.

Outstandingly, western blot analysis displayed that vitamin C (5mM) treatment almost completely abolished ERK1/2 phosphorylation in both, SW480 and LoVo CRC lines without affecting ERK1/2 expression. Moreover, phosphorylation of ERK1/2 was hardly observable in normal human colonocytes (HCEC) with wild type KRAS (Figure 2A) after 20hr treatment. Dramatic inhibition of ERK1/2 phosphorylation was confirmed *in vivo* by immunohistochemistry assays in vitamin C-treated SW480 xenografts (Figure 4C). generated in athymic nude mice Foxn1nu.

Upwards in the phosphorylation cascade, mitogen-activated protein kinase kinase 1 (MEK1) expression and phosphorylation was also observed to be downregulated by vitamin C treatment in both cell lines tested. On the other hand, although MEK1 phosphorylation was detected in normal HCEC, neither protein expression nor phosphorylation were affected by vitamin C (Figure 2B).

The first step in the EGFR/MAPK phosphorylation cascade is the activation of KRAS. We wondered if vitamin C could target KRAS straightly in a way that could explain the alterations observed in ERK1/2 and MEK1 phosphorylation state.

To our surprise, double immunofluorescence assays carried out in LoVo cells using antibodies raised against human RAS and cytokeratin 20, displayed KRAS mislocalization from plasma membrane and colocalization with cytoplasmic cytokeratin (Figure 2C) after vitamin C treatment.

It is known that Ras proteins regulate signaling pathways important for cell growth, differentiation, and survival. Constitutive KRAS signaling activity depends on its level of enrichment on the plasma membrane [18]. As described, Ras trafficking to the cell plasma membrane is a multi-step process involving farnesylation of the cysteine residue of the RAS CAAX motif, methylation of the α-carboxyl group and finally, Ras proteins take one of two possible routes to the cell surface [19, 20].

So, we observed that vitamin C was able to induce RAS detachment from cell plasma membrane, but the question regarding the molecular mechanism underlying this observation needs to be addressed.

In 2010, Weinberg F *et al*., reported that mitochondrial metabolism allows for the generation of reactive oxygen species (ROS) which are required for Kras-induced anchorage-independent growth through regulation of the ERK/MAPK signaling pathway [21].

As it has been previously described, vitamin C is a potent intracellular ROS quencher [22]. So we performed assays using dichlorofluorescin diacetate (DCFDA), a fluorogenic dye that measures hydroxyl, peroxyl and other reactive oxygen species (ROS) activity within the cell, in order to check out ROS levels in SW480 and LoVo cells after vitamin C treatment. Interestingly, we detected that vitamin C treatment dramatically reduced intracellular ROS levels in SW480 and LoVo cells (Figure 2D), thus vitamin C-ROS quenching hampers KRAS trafficking to the cell plasma membrane, then interfering with downstream RAS/ERK phosphorylation cascade.

### Vitamin C downregulates the Warburg checkpoints GLUT-1 and PKM2 through inhibition of PKM2 phosphorylation at Ser 37

Malignant transformation of a normal cell into a cancer cell invariably correlates to metabolic alterations and high glucose intake. The Glucose transporter 1 (GLUT1) catalyzes facilitative diffusion of glucose into the normal cell and it is often upregulated in many tumors fulfilling the high glucose requirements. Interestingly, GLUT-1 is considered as a master regulator of the Warburg effect during the neoplastic transformation [23]. GLUT-1 can be employed as a molecular marker in CRC to indicate the tumor hypoxia degree [24], HIF-1 expression and resistance to chemotherapy [25, 26].

Therefore we aimed to check out GLUT-1 status in human colon cancer and to observe if vitamin C could be able to regulate GLUT-1 expression in human KRAS mutant cancer cells and tumors.

Immunohistochemistry (IHC) assays carried out in normal human colon mucosa (upper and bottom crypts) and colon adenocarcinoma showed strong GLUT-1 upregulation in CRC (Figure 4A). Interestingly, in the same experiment we did not detect tumor overexpression of vitamin C receptor SLC23A1.

**Figure 3:**
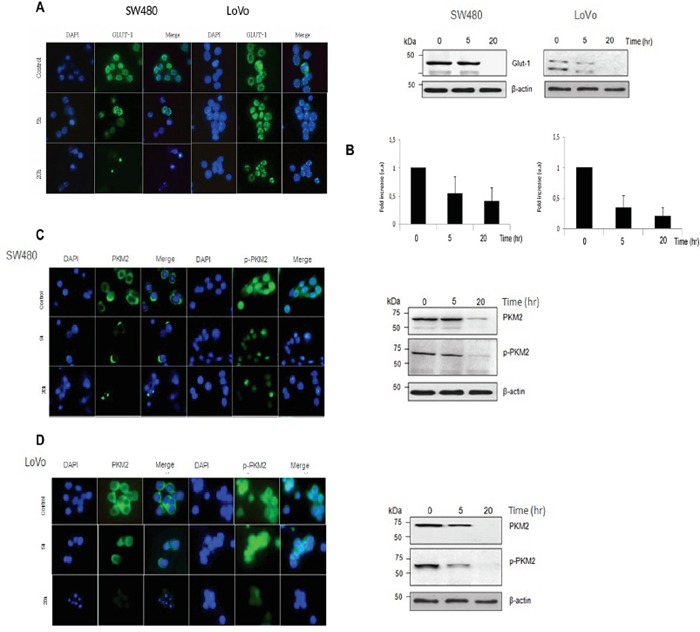
Vitamin C impairs the Warburg effect in *KRAS* mutant cells through downregulation of GLUT-1 and PKM2 **A.** immunofluorescence analysis of GLUT-1 expression in SW480 and LoVo cells after vitamin C treatment (8 mM) for 20 hr. Glucose receptor 1 (GLUT-1) is shown in green. Cell nucleus is depicted in blue after DAPI staining. **B.** western blot and Real Time Quantitative PCR analysis of GLUT-1 protein and mRNA expression in SW480 and LoVo cells after vitamin C treatment for 20 hr. **C.** immunofluorescence analysis of PKM2 and p-PKM2 expression in SW480 and cells after vitamin C treatment (5 mM) for 20 hr. PKM2 and p-PKM2 is shown in green. Cell nucleus is depicted in blue after DAPI staining. Western-Blot analysis of PKM2 and p-PKM2 expression in SW480 cells after vitamin C treatment for 20 hr. **D.** immunofluorescence analysis of PKM2 and p-PKM2 expression in LoVo cells after vitamin C treatment (8 mM) for 20 hr. PKM2 and p-PKM2 is shown in green. Cell nucleus is depicted in blue after DAPI staining. Western-Blot analysis of PKM2 and p-PKM2 expression in LoVo cells after vitamin C treatment for 20 hr.

**Figure 4:**
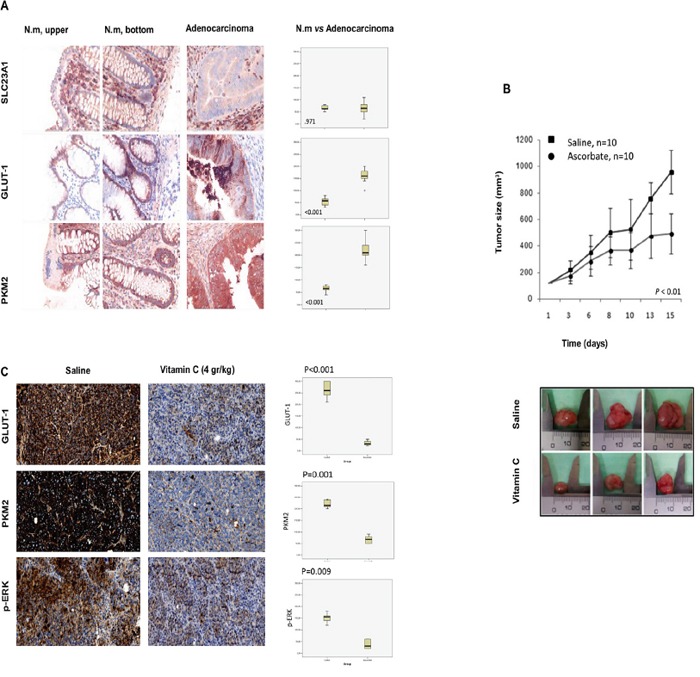
Vitamin C inhibits tumor growth in SW480 xenografts generated in athymic nude mice Foxn1nu though downregulation of GLUT-1, p-ERK and PKM2 **A.** immunohistochemistry carried out in human normal colon mucosa (upper and bottom) and colon adenocarcinoma shows differential expression of GLUT-1 and PKM2. Vitamin C receptor (SLC23A1) shows no differential expression. Box-plot shows the relative expression of SLC23A1, GLUT-1 and PKM2 in normal colon mucosa and adenocarcinoma (upper and bottom crypt). Box represents 25th and 75th percentile expression and bar representsmedian. Whiskers corresponds to 5th and 95th percentile and outliers aremarked separately. **B.** tumor growth of SW480 KRAS (G12V) xenograft in female athymic nude mice (Foxn1nu, 7 weeks old). Suspensions of 2×10^6^cells were injected subcutaneously in control and experimental mice. Animals were treated (*i.p*) with vehicle or vitamin C (4 gr/kg body weight) for 15 days once daily. Tumors were measured twice weekly in three perpendicular dimensions using a Vernier caliper and tumor volume was calculated. **C.** immunohistochemistry assays in SW480 tumors shows *in vivo* downregulation of PKM2, GLUT-1 and p-ERK in vitamin C-treated tumors. Box-plot shows the relative expression of GLUT-1, PKM2 and p-ERK in normal colon mucosa and adenocarcinoma (upper and bottom crypt). Box represents 25th and 75th percentile expression and bar Whiskers correspond to 5th and 95th percentile and outliers are marked separately.

SW480 and LoVo cancer cells were treated with pharmacological doses of vitamin C (5 mM) for 5 and 20 hr and dramatic downregulation of GLUT-1 protein was observed as showed in Western Blot and immunofluorescence assays (Figure 3A). Experiments performed using Quantitative PCR showed also significative downregulation of GLUT-1 mRA pointing out to a transcriptional (direct or indirect) regulation of GLUT-1 by vitamin C (Figure 3B). Impressive inhibition of GLUT-1 expression in tumors was also observed *in vivo* in vitamin C-treated murine SW480 xenografts (Figure 4C). Therefore, data obtained strongly supports that vitamin C targets Warburg metabolism and it is able to interfere with the expression of crucial checkpoints in the neoplastic transformation.

However, in order to establish a molecular mechanism it is necessary a link between the effects of vitamin C in the MAPK/EGFR pathway and the observed GLUT-1 downregulation.

In 2012, Yang W *et al*., reported that aberrant EGFR pathway activation in human cancer cells induces PKM2 nuclear translocation, which is mediated by Pyruvate kinase M2 (PKM2) phosphorylation at Ser37 by ERK. PKM2 (Ser37) is shuttled into the cell nucleus acting as a coactivator of *β*-catenin, inducing c-Myc expression which in turn upregulates the expression of glycolytic enzymes such as GLUT1 and LDHA [27].

PKM2 is upregulated in CRC providing tumor growth advantages [28, 29] contributing to gefitinib resistance via upregulation of STAT3 activation in colorectal cancer [30]. Therefore, PKM2 can be considered as a potential therapeutic target in colon and pancratic neoplasias.

In IHC assays we could confirm that PKM2 is upregulated in colon cancer when compared with normal mucosa (Figure 4A), therefore we aimed to check out any putative effect of vitamin C in PKM2 expression and phosphorylation in Ser 37 that could link GLUT-1 downregulation with the observed inhibition of the MAPK/EGFR pathway.

Outstandingly, western blot analysis displayed inhibition not only of PKM2 but also p-PKM2 (ser37) in both KRAS mutant SW480 and LoVo cells (Figure 3C–3D) and this observation could be confirmed in immunofluorescence assays using antibodies raised against PKM2 and p-PKM2 (ser37). Interestingly, in this occasion mRNA expression of PKM2 appeared to be unaffected by ascorbate treatment (data not shown).

We could also observe an *in vivo* PKM2 drastic inhibition in murine SW480 xenografts, thus correlating with reduced tumor growth after intraperitoneal vitamin C treatment.

Downregulation of GLUT-1 expression and PKM2 expression and phosphorylation at ser37 strongly corroborates the initial hypothesis, stating that vitamin C exerts its selective antitumoral activity targeting enzymes, signaling pathways and metabolic processes involved in the Warburg effect.

### Physiological concentrations of vitamin C inhibits KRAS mutant colonosphere-formation downregulating c-Myc

Colonospheres derived from colon cancer cell lines usually show increased levels of total *β*-catenin inducing TCF/LEF transactivation and c-Myc overexpression that confers molecular features associated with resistance to drugs, radioresistance and poor clinical outcome [31].

Strikingly, vitamin C treatment on colonospheres derived from SW480 and LoVo CRC cell lines showed a drastic effect not only in colonosphere formation, but also in the number of cell per single sphere in both cases (Supplementary Figure 1A and 1B). However the most noticeable result is that the effect was observed at previously described human physiological concentrations of vitamin C (≤ 200 μM).

Interestingly, western blot analysis showed strong inhibition of c-Myc oncogene in colonospheres treated at concentrations of vitamin C as low as 100 μM, suggesting that vitamin C also could be interfering with the constitutively activated WNT pathway in colon cancer (Supplementary Figure 1C).

As it was said previously, there is a strong interplay between the two major signaling routes altered in colon cancer: EGFR/MAPK and WNT signaling pathway. We do think that vitamin C inhibition of PKM2 phosphorilation at serine 37 avoids PKM2 translocation to the cell nucleus, therefore stalling *β*-catenin-TCF/LEF dependent GLUT-1 and c-Myc expression. In spite of this, straight activity of vitamin C in the regulation of WNT pathway partners cannot be ruled out.

### PKM2 inhibition is mediated by vitamin C downregulation of polypyrimidine tract binding protein 1 (PTB1)

However, results obtained are not enough to explain the previously observed effect of vitamin C in PKM2 protein dowregulation.

Polypyrimidine Tract Binding Protein 1 (PTB1) belongs to the subfamily of heterogeneous nuclear ribonucleoproteins (hnRNPs) and has been reported to regulate PKM2 alternative mRNA splicing [32]. IF and western blot assays displayed that PTB expression was clearly inhibited after 20 hr of vitamin C (5mM) after treatment (Supplementary Figure 2A and 2C) although no effect was observed at mRNA level (Supplementary Figure 2B). Interestingly, PTBP1 expression is controlled by the complex formed by β-catenin, TCF/LEF and nuclear PKM2 (Ser37). We have observed that vitamin C is capable to stall phosphorylation of PKM2 at Ser 37 then involving PTBP1 downregulation and therefore hampering PKM2 mRNA alternative splicing.

Summarizing the overall data here reported, we describe a novel molecular mechanism of vitamin C that presents the Warburg metabolism and related MAPK/ EGFR pathway as primary targets of vitamin C in colon cancer.

**Figure 5:**
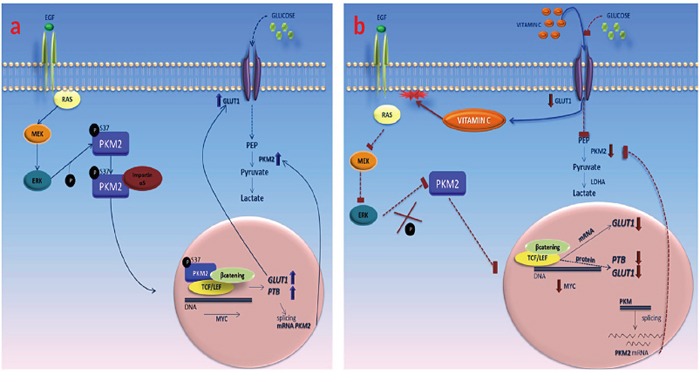
Vitamin C impairs the Warburg effect in KRAS mutant cells through downregulation of GLUT-1 and PKM2 **A.** in absence of vitamin C, PKM2 is phosphorylated at ser 37 and translocates to the cell nucleus. Then, p-PKM2 binds to β-catenin and TCF/LEF transcriptional complex promoting c-Myc transcription that, in turn enhances the expression of GLUT-1 and PTB that participates in the splicing of PKM2 mRNA. **B.** vitamin C enters into the cell via GLUT-1 and SVCT1 inducing RAS detachment from plasma membrane, blocking downstream phosphorylation of PKM2 phosphorylation at ser 37. Disruption of the transcriptional complex formed by p-PKM2, β-catenin and TCF/LEF leads to downregulation of c-Myc, GLUT-1 and PTB expression. Absence of PTB activity stalls the splicing of PKM2 mRNA.

As it is shown in Figure 5, vitamin C (ascorbic and dehydroascorbic acid) enters to the cancer cell quenching reactive oxygen species (ROS) and therefore inducing RAS detachment from plasma membrane. As a consequence of RAS mislocation, EGFR/MAPK phosphorylation cascade is stalled, thus inhibiting PKM2-ERK1/2 dependent phosphorylation at Serine 37. Absence of p-PKM2 nuclear translocation inhibits GLUT-1 and PKM2-PTB dependent expression. The final result is a vitamin C selective killing effect in colon cancer cells.

The presented model also raises a provocative question: may vitamin C (and / or other molecules targeting Warburg key players) open the door for anti-EGFR therapies in *KRAS/BRAF* mutant patients? We do think that further research and clinical assays addressing this query are essential to boost the quest for new anticancer strategies.

## MATERIALS AND METHODS

### Cell culture and cell lines

SW480, LoVo and RKO cells were cultured in DMEM supplemented with 10% fetal calf serum (FCS) (both from Invitrogen). Cell lines were originally obtained from the American Type Culture Collection (ATCC) and authenticated using the GenePrint® 10 System (Promega), which allows co-amplification and three-color detection of ten human loci: TH01, TPOX, WA, Amelogenin, CSF1PO, D16S539, D7S820, D13S317, D21S11 and D5S818. Short Tandem Repeats profiles were sent for comparison against cell line datebases (ATCC, DSMZ). Last test was done on February 2015. Immortalized human colonocytes (HCEC) were provided by Prof. Manel Esteller IDIBELL (Barcelona, Spain).

### Antibodies

GLU-1 antibody (1:5000, Millipore, Cat. #: 07-1401), PKM2 (Ser37) antibody (1:1000, Cell Signaling, Cat. 11456), p-ERK1/2 antibody (1: 100, Abcam, Cat. ab24157) were employed for immunohistochemistry assays.

Anti-Glut-1 antibody (1:200, Abcam, Cat. ab137656), MEK1 antibody (1:1000, Abcam, Cat. ab96379), ERK1/2 antibody (1:2000, Thermofisher, Cat. ERK-7D8), p-ERK1/2 antibody (1:1000, Abcam, Cat. ab24157), MAPK (p44/42) antibody (1:500, Cell Signaling, Cat. #9102), c-Myc antibody (1:500, Abcam, Cat. ab32) and Beta-actin antibody (1:10.000, Cell signaling, Cat. #4967) were used for western blot and immunofluorescence assays.

### Cell viability assays

HCEC, LoVo and SW480 cells were treated with pharmacological concentrations of vitamin C (0-10mM) for 2h, and then replaced with normal DMEM supplemented with 10% fetal calf serum (FCS). For time dependent viability assays, LoVo and SW480 cells were treated withvitamin C (10 mM) for 2h and then collected (0, 5, 12, 36 h) for cell counting using a TC20™ Automated Cell Counter (Biorad). For cetuximab and vitamin C combinatorial assays, HT29 harboring wild type *KRAS*, and the *KRAS* mutant LoVo and SW480 were treated with cetuximab (calculated IC50=0,4 μM), vitamin C (5mM) alone and combination for 12 hr. Then, cells were tripsinized and fixed with trypan blue solution (Sigma-Aldrich). Cell counting was carried out using a TC20™ Automated Cell Counter (Biorad).

### Reactive oxygen species (ROS) measurement

Activity of vitamin C in quenching intracellular reactive oxygen species (ROS) was measured using a Cellular ROS/Superoxide Detection Assay Kit (Abcam). SW480 and LoVo CRC lines were exposed to ascorbate (7 mM) and PBS for controls for 20 hr. After treatment, cells were washed twice with PBS solution and then incubated in medium containing 10 μmol/l H2DCF-DA at 37°C for 30 minutes. After incubation, cells were harvested by trypsinization, and intracellular levels of ROS were measured by fluorescence emission with a 1260 InfinityFluorescence Detector (Agilent). Relative intracellular ROS levels after treatment with vitamin C were calculated in relation to control, which was given a value of 100.

### Apoptosis measurements

HCEC, LoVo and SW480 cells were treated with vitamin C (10mM) for 2h, and then replaced with normal DMEM supplemented with 10% fetal calf serum (FCS). 1–5 × 10^5^ cells were collected by centrifugation and resuspended in 500 μL of 1X binding buffer. Afterwards, 5 μL of annexin V-FITC and 5 μL of propidium iodide were added and incubated for 5 min in the dark. V-FITC binding was analyzed by flow cytometry using a BD FACSCanto II device (Becton, Dickinson and Company) (Ex = 488 nm; Em = 350 nm) using FITC signal detector and PI staining by the phycoerythrin emission signal detector.

### Immunofluorescence assays

For immunofluorescence analyses, cells were rinsed once in PBS, fixed in 3.7% paraformaldehyde for 15 min at RT and rinsed once in 0.1 M glycine and twice in PBS. They were permeabilized in 0.5% Triton X-100 and then washed three times in PBS. The non-specific sites were blocked by incubation with PBS containing 1% goat serum for 30 min at RT. Next, cells were incubated with a rabbit or goat polyclonal antibodies against PKM2 (Abcam, ab38237), PKM2 (phospho-Ser37) (Signalway Antibody, 11456), GLUT-1 (Abcam, ab652), SLC23A1 (Abcam, ab112912), PTB (PTBP1, ab5642), Ras antibody [F132-62] (Abcam, ab16907) and cytokeratine 20diluted in PBS for 3 h at RT or overnight at 4°C. After four washes in PBS, cells were incubated with secondary antibodies for 45 min at RT, washed and mounted in VectaShield (Vector Laboratories). Confocal microscopy was performed with a LSM510 laser scanning microscope (Carl Zeiss) equipped with argon (488 nm), HeNe (543 nm) and HeNe (633 nm) ion lasers. All confocal scans were acquired with the LSM510 software using a Plan Apochromat 63x NA 1.4 objective (Carl Zeiss). For double or triple labeling experiments, images of the same confocal plane were sequentially recorded and pseudocolor images were generated and superimposed.

### Immunohistochemical analysis

Staining of human tissues was performed as described (31). The paraffin embedded sections were cleared and the sections were incubated with 0.1% Pronase (Roche #165 921) in 0.1% CaCl2 pH 7.8. at 37C for 10 minutes. They were blocked with 3% H2O2 in TBS for 10 mins., washed then blocked with Dako Biotin Blocking System (Dako X0590). After washing, they were further blocked with 10% Normal Rabbit Serum for 10 mins at room temperature (RT) and incubated firstly with p-ERK (Abcam, ab65142) dilution 1:50, PKM2 (Abcam, ab38237) dilution 1:100 and GLUT-1 (Abcam, ab652) dilution 1: 100for 1hr at RT, then with biotinylated Rabbit Anti-Mouse (Dako, E-0354) at 1/100 for 30 mins. at RT, and finally with Strep-ABC complex (Dako, K-0377) at 1/100 for 30 mins. at RT. The sections were developed with AEC substrate kit (vector lab, SK-4200) at RT for 20 mins., counterstained with haematoxylin and mounted with DAKO aqueous mount (Dako, 003181).

### Colonosphere formation assay

For the colonosphere formation assay, after treatment with different doses of vitamin C for 48 h, cells were tripsinized, counted and re-seeded at clonal density (1 cell/μl) in 96-well plate with ultra-low attachment surface (Costar, Corning, NY, USA) with serum free Dulbecco's MEM Nutrient Mixture F+12 Ham medium supplemented with 10 ng/ml basic fibroblast growth factor, 20 ng/ml epidermal growth factor and 1% v/v methylcellulose (R&D Systems, Minneapolis, MN, USA) to prevent cell aggregation. The supplements were freshly added every 2–3 days and the number and size of formed colonospheres were evaluated by optical microscopy on day 7 after seeding. Secondary colonospheres were formed from the cell population obtained after trypsin-EDTA disaggregation of primary spheres and seeded at clonal density and cultured as described above. To obtain a sufficient cell number for secondary colonosphere formation, primary colonospheres were seeded for 7 days in 6-well ultra-low attachment plates.

### Murine assays

SW480 *KRAS* (G12V) cells were used to generate xenograft model in female athymic nude mice Foxn1nu, 7 weeks old (Harlan Laboratories). Suspensions of 2×10^6^cells were injected subcutaneously in control and experimental mice. Once tumor size volume reached 100 mm^3^mice were treated with intraperitoneal (*i.p*) vehicle or vitamin C (4gr/Kg) once daily with the same dosing schedule (SW480: n = 10) for 15 days. Tumors were measured twice weekly in three perpendicular dimensions using a vernier caliper and tumor volume was calculated using the ellipsoidal formula V (mm^3^) = 1/6 π x length (mm) x width^2^ (mm^2^)

### Patients

Formalin-fixed paraffin-embedded 3 μm tissue sections from human non-pathological colon (n = 16) and small intestine (n = 16), colorectal adenomas (n = 76) and primary tumors from metastatic colorectal (mCRC) patients (n = 699) were retrieved from Fundación Jiménez Díaz Biobank. Tumor specimens were retrospectively selected from consecutive mCRC patients (1998-2009), which had fulfilled the following criteria: adenocarcinoma, metastatic disease, no neoadjuvant therapy, available tissue and clinical follow up. Clinical data and follow up were collected from medical clinical records by medical oncologists. The study was approved by the Ethics Committee of the institution (PIC 23/2012).

## SUPPLEMENTARY FIGURES


