# Supplementary material for: Vitamin C uncouples the Warburg metabolic switch in KRAS mutant colon cancer
Source: Oncotarget. 2016 Jun 15;7(30):47954–65. doi: 10.18632/oncotarget.10087 (PMC5216991; doi:10.18632/oncotarget.10087)
Supplement: Supplementary file 1 [file oncotarget-07-47954-s001.pdf]

Vitamin C uncouples the Warburg metabolic switch in KRAS mutant colon cancer

SUPPLEMENTARY FIGURES

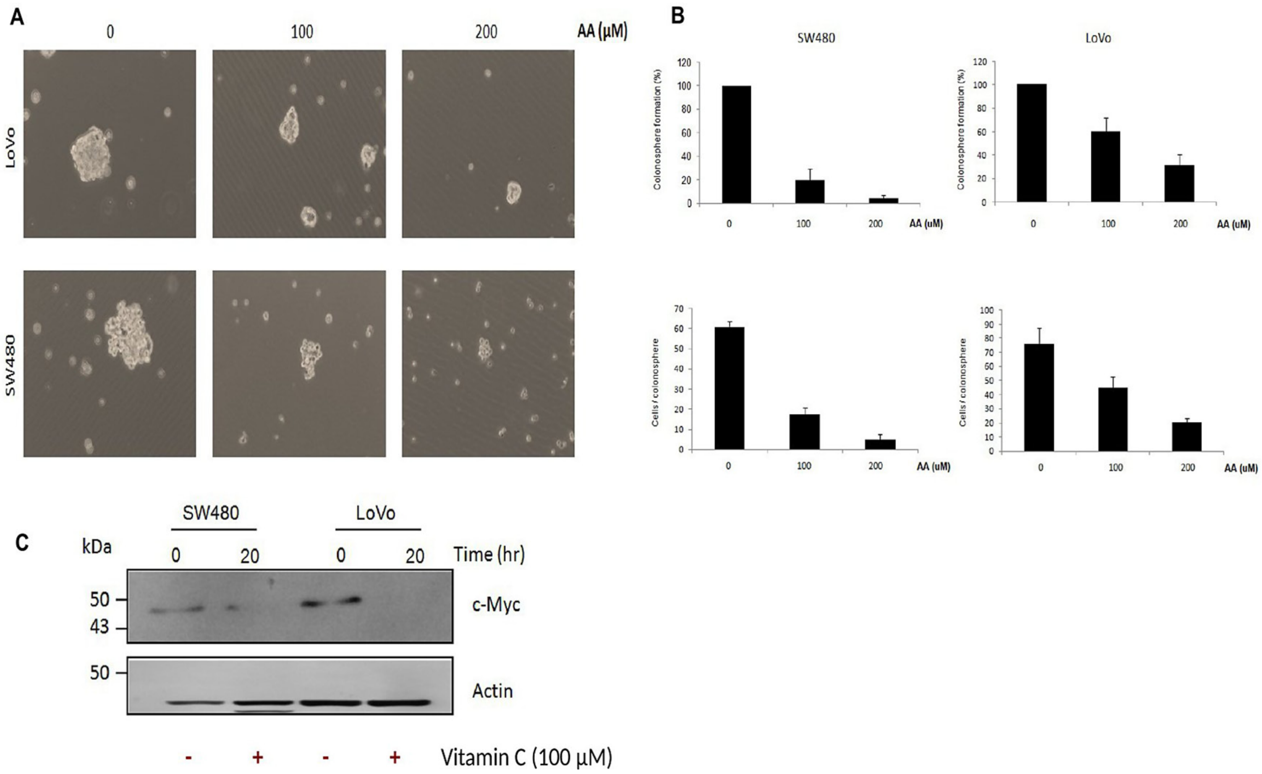

Supplementary Figure S1: Physiological concentration of vitamin C is able to inhibit the formation of colonospheres derived from *KRAS* mutant CRC lines through downregulation of c-Myc. A. representative picture depicting inhibition of colonosphere formation in SW480 and LoVo cells treated with physiological concentrations of vitamin C (100-200 µM) for 7 days. **B.** quantification of the effect of physiological vitamin C (100 µM) on SW480 and LoVo colonospheres formation and number of cells per sphere after 7 days of treatment. One-way ANOVA followed by Dunnett's post-test for multiple comparisons. *p< 0.05, **p < 0.001, n = 3. **C.** western blot analysis shows inhibition of c-Myc expression in colonospheres treated with vitamin C (100 µM) for 7 days.

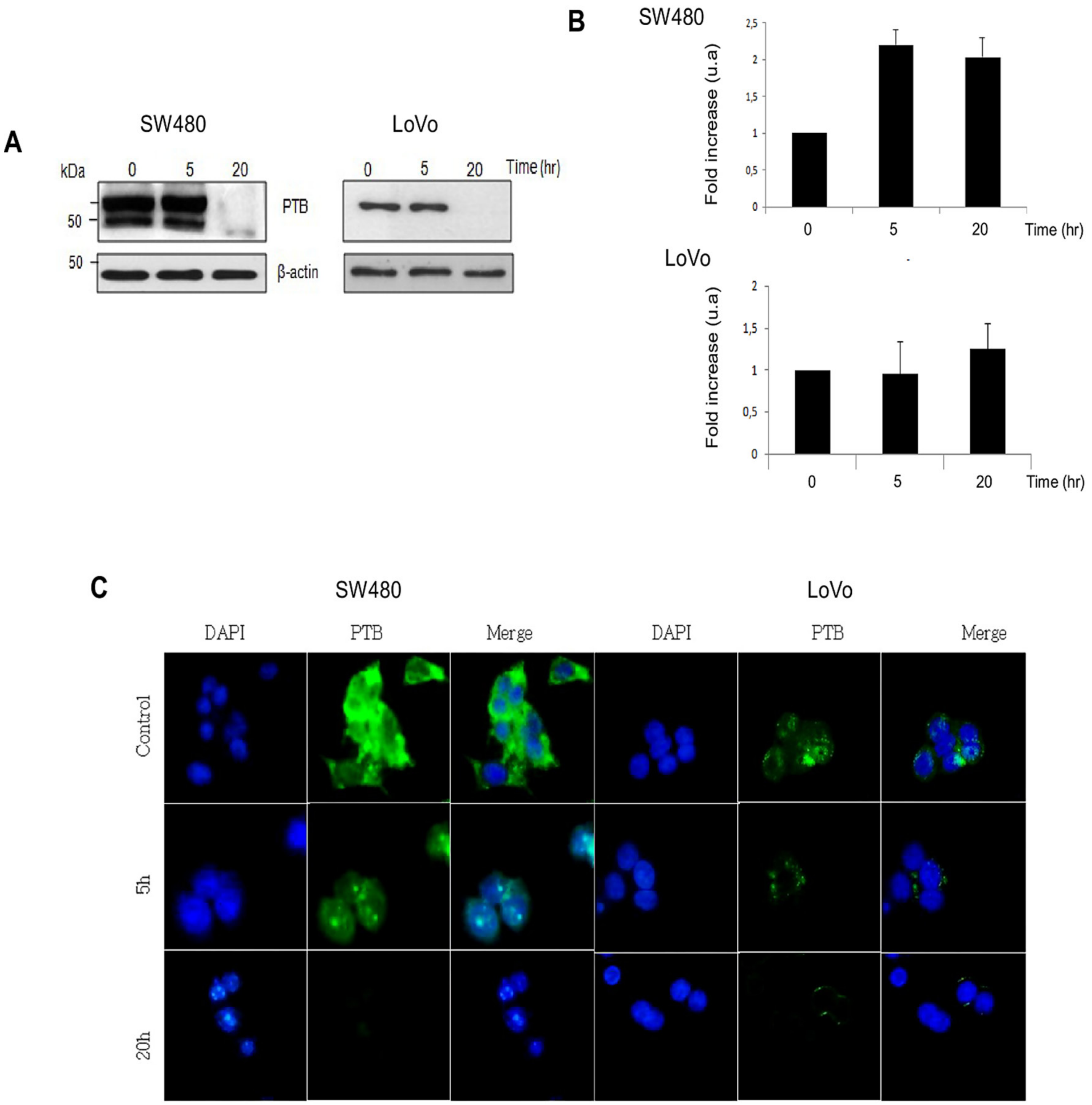

Supplementary Figure S2: Vitamin C downregulates Polypyrimidine tract 1 binding protein (PTB1). A. western-Blot of PTB1 in SW480 and LoVo cells after vitamin C treatment for 20 hr. **B.** real Time Quantitative PCR analysis expression shows no decreaseof PTBP1 mRNA in SW480 and LoVo cells after vitamin C treatment for 20 hr. **C.** immunofluorescence analysis of PTB1 expression in SW480 and LoVo cells after vitamin C treatment (8 mM) for 20 hr.
